# Supplementary material for: Genome-Wide Identification, Evolution and Expressional Analysis of OSCA Gene Family in Barley (Hordeum vulgare L.)
Source: Int J Mol Sci. 2022 Oct 27;23(21):13027. doi: 10.3390/ijms232113027 (PMC9653715; doi:10.3390/ijms232113027)
Supplement: Supplementary file 1 [file ijms-23-13027-s001.zip › Supplementary file 1.pdf]

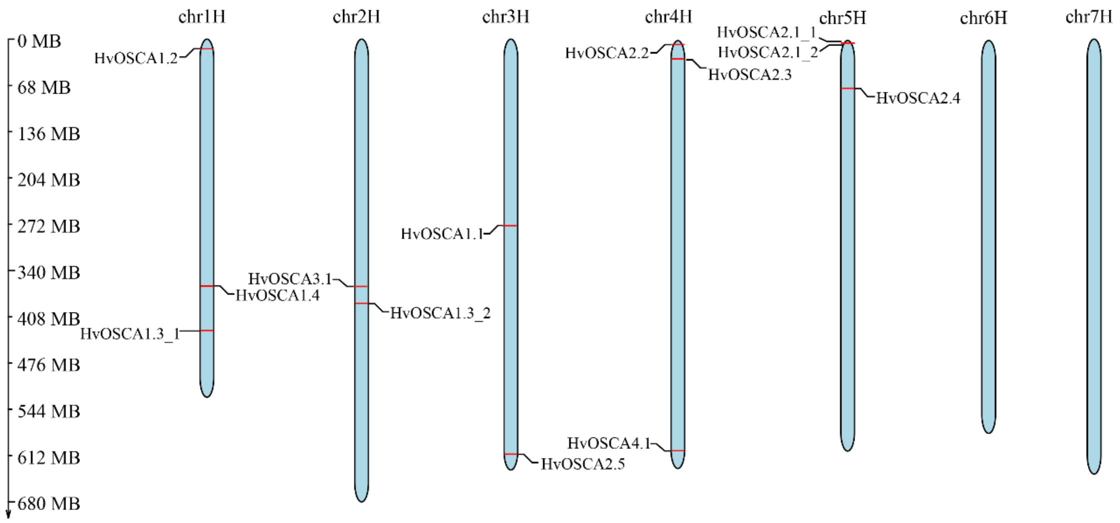

Figure S1. The schematic visualized physical position of *HvOSCA*s and its distribution on barley chromosomes.

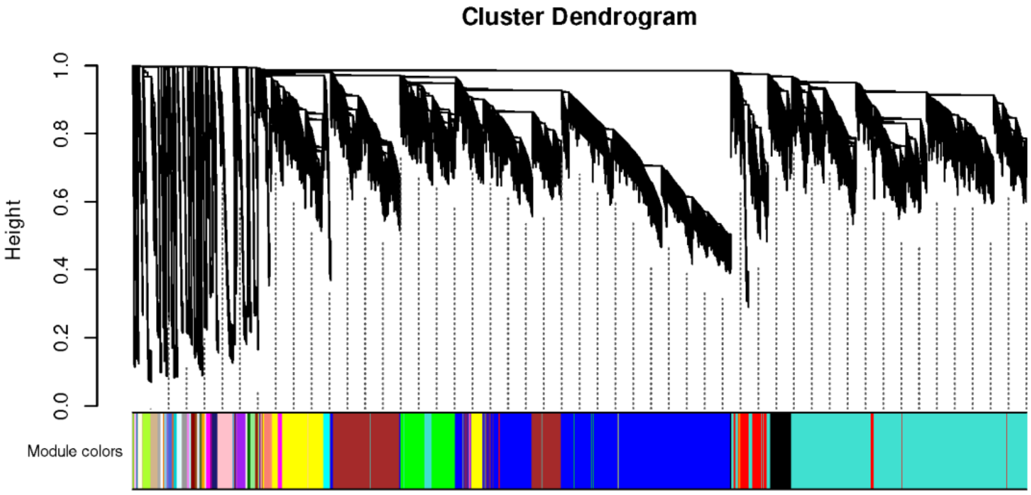

Figure S2. Weighted gene co-expression network analysis (WGCNA) of *HvOSCA*s under stress treatments

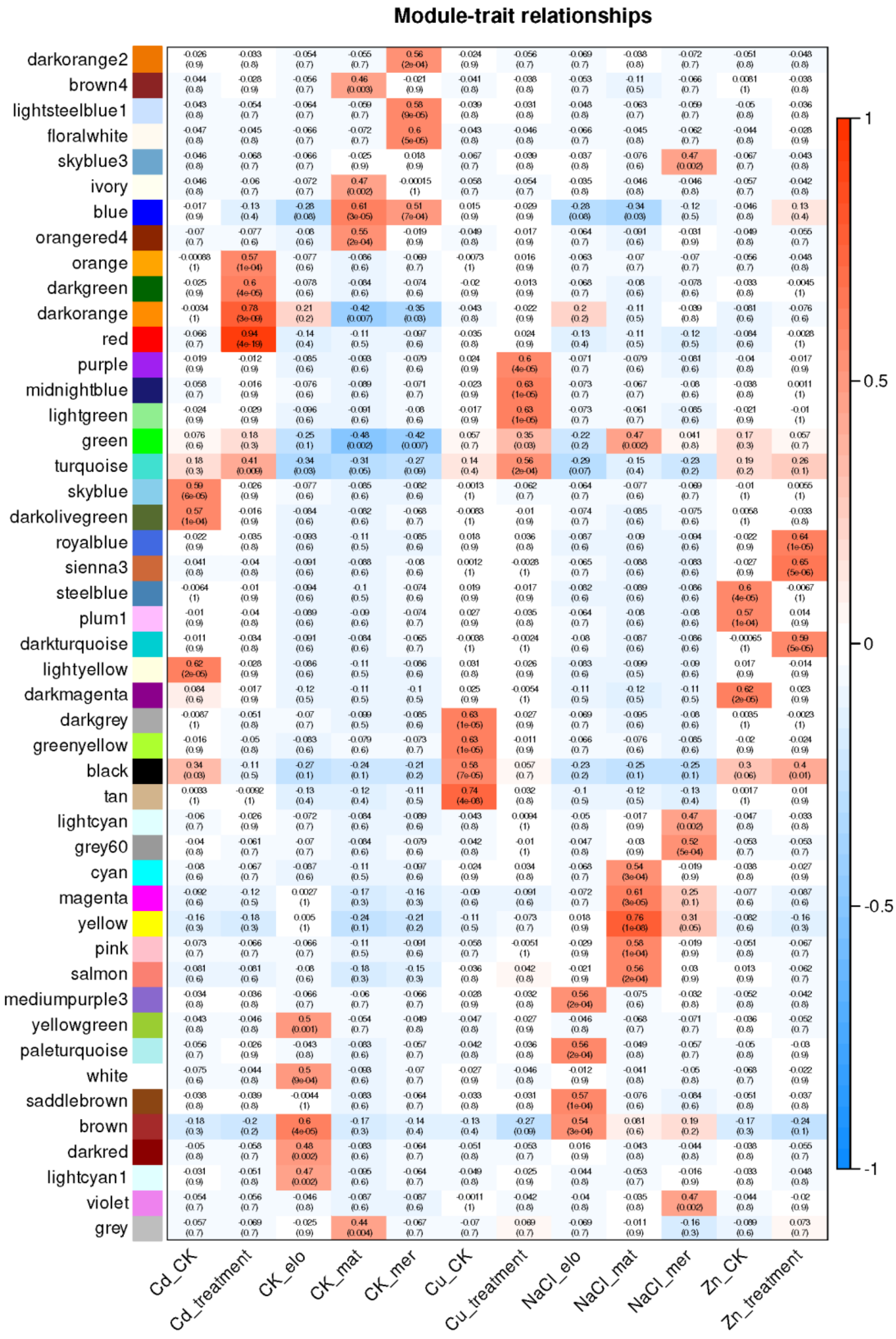

Figure S3. The heat map of module-treatment relationship. Each row represented a module eigengene, column represent a treatment. Each cell contains two data up for correlation coefficient and bottom for p-value. The heat map was coded by correlation

coefficient from -1 to 1 according color legend on the right. Simulated abiotic stress treatment include cold, salinity, and heavy metal ion treatment. cold stress treatment: CK of cold(cold_CK), cold (4°C) treatment (Cold_treatment), salt treatment of *H.vulgare* root (dissected and sampled the *H.vulgare* root according the organizational structure for meristem zone, elongation zone and mature zone). CK of meristem zone (CK_mer), meristem zone under salt treatment (NaCl_mer), CK of elongation zone (CK_elo), elongation zone under salt treatment (NaCl_elo), CK of maturation zone (CK_mat) and maturation zone under salt treatment (NaCl_mat). Heavy metal ion (cadmium ion, copper ion and zinc ion) stress. control of cadmium ion stress (Cd_ck), cadmium ion treatment (Cd_treatment), control of copper ion treatment (Cu_CK), copper ion treatment (Cu_treatment), control of zinc ion stress (Zn_CK) and zipper ion treatment(Zn_treament).

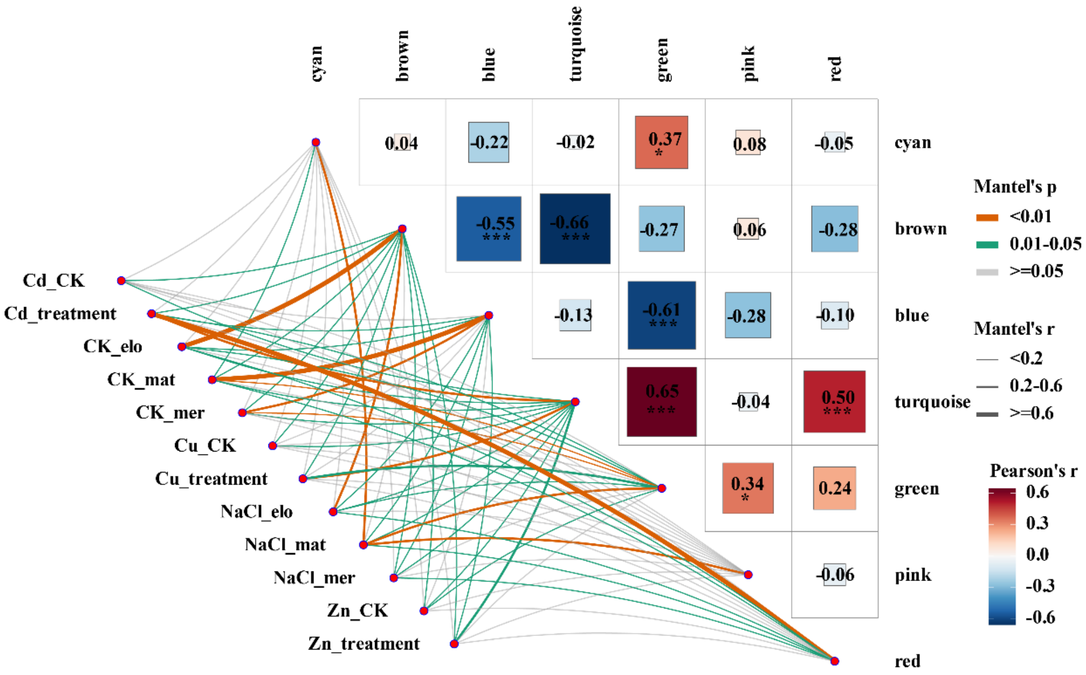

Figure S4. Correlations between 7 modules and simulated abiotic stresses treatment included cold, salinity, and heavy metal ion treatment. cold stress treatment: CK of cold(cold_CK), cold (4°C) treatment (Cold_treatment), salt treatment of *H.vulgare* root (dissected and sampled the *H.vulgare* according the organizational structure of root for meristem zone, elongation zone and mature zone). CK of meristem zone (CK_mer), meristem zone under salt treatment (NaCl_mer), CK of elongation zone (CK_elo), elongation zone under salt treatment (NaCl_elo), CK of maturation zone (CK_mat) and maturation zone under salt treatment (NaCl_mat). Heavy metal ion (cadmium ion, copper ion and zinc ion) stress. control of cadmium ion stress (Cd_ck), cadmium ion treatment (Cd_treatment), control of copper ion treatment (Cu_CK), copper ion treatment (Cu_treatment), control of zinc ion stress (Zn_CK) and zipper ion treatment (Zn_treament).
