# Supplementary material for: Genome-Wide Identification, Evolution and Expressional Analysis of OSCA Gene Family in Barley (Hordeum vulgare L.)
Source: Int J Mol Sci. 2022 Oct 27;23(21):13027. doi: 10.3390/ijms232113027 (PMC9653715; doi:10.3390/ijms232113027)
Supplement: Supplementary file 1 [file ijms-23-13027-s001.zip › Figures.zip!/Figure S1.pdf]

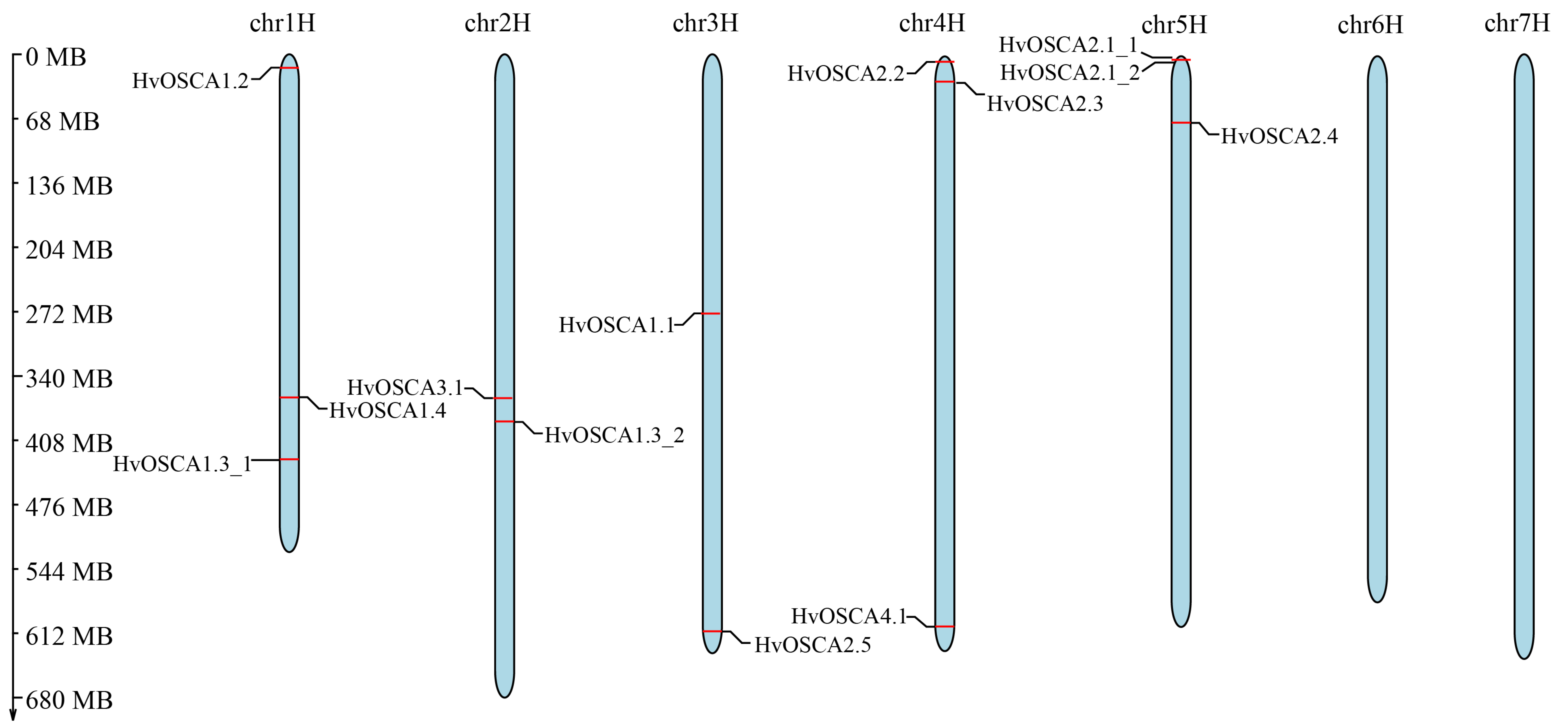
chr1H
chr2H
chr3H
chr4H
chr5H
chr6H
chr7H
0 MB
68 MB
136 MB
204 MB
272 MB
340 MB
408 MB
476 MB
544 MB
612 MB
680 MB
HvOSCA1.2
HvOSCA1.4
HvOSCA1.3_1
HvOSCA3.1
HvOSCA1.3_2
HvOSCA1.1
HvOSCA2.5
HvOSCA2.2
HvOSCA2.3
HvOSCA4.1
HvOSCA2.1_1
HvOSCA2.1_2
HvOSCA2.4
