# Supplementary material for: Genome-Wide Identification, Evolution and Expressional Analysis of OSCA Gene Family in Barley (Hordeum vulgare L.)
Source: Int J Mol Sci. 2022 Oct 27;23(21):13027. doi: 10.3390/ijms232113027 (PMC9653715; doi:10.3390/ijms232113027)
Supplement: Supplementary file 1 [file ijms-23-13027-s001.zip › Figures.zip!/Figure S2.pdf]

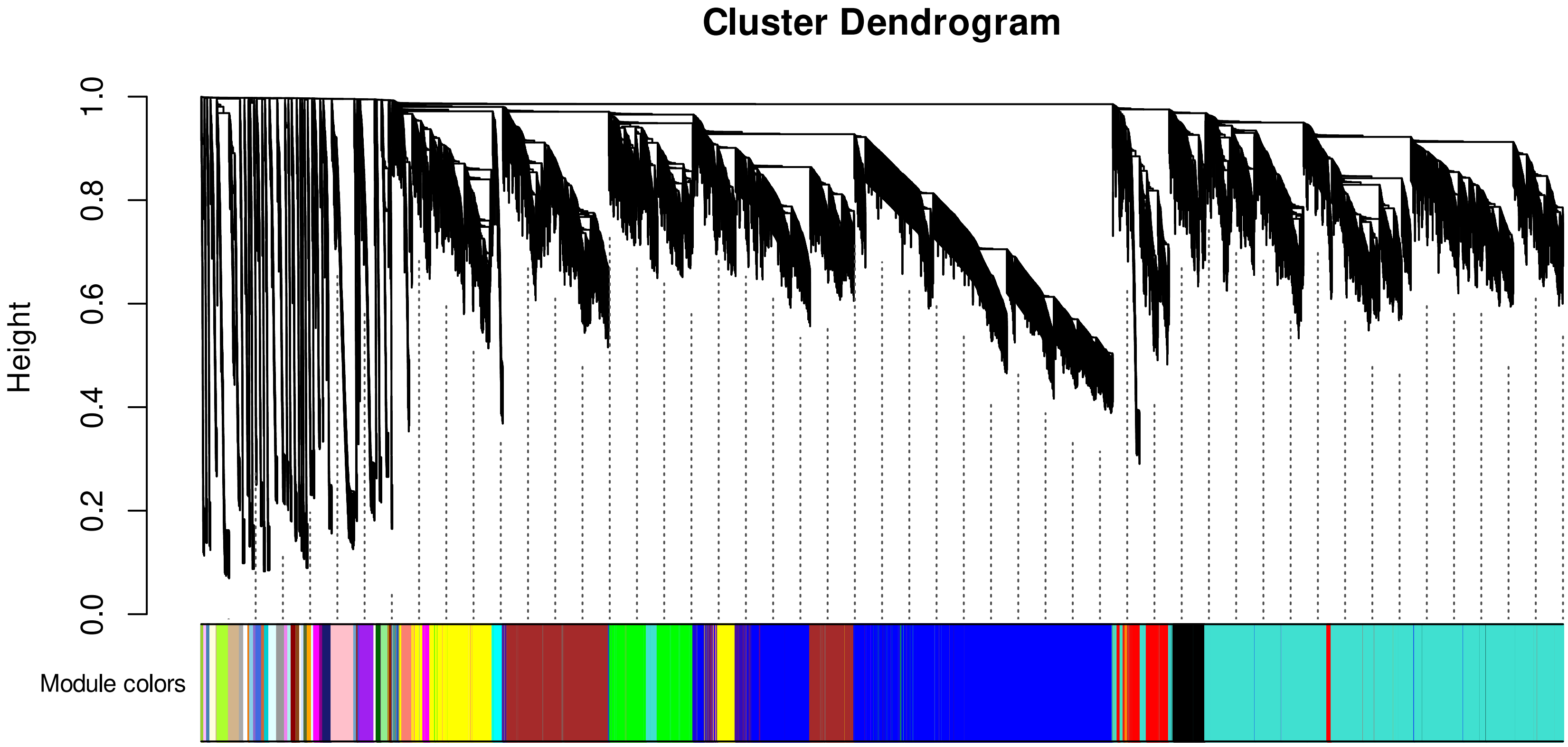
Cluster Dendrogram
Height
1.0
0.8
0.6
0.4
0.2
0.0
Module colors
