# Supplementary material for: Genome-Wide Identification, Evolution and Expressional Analysis of OSCA Gene Family in Barley (Hordeum vulgare L.)
Source: Int J Mol Sci. 2022 Oct 27;23(21):13027. doi: 10.3390/ijms232113027 (PMC9653715; doi:10.3390/ijms232113027)
Supplement: Supplementary file 1 [file ijms-23-13027-s001.zip › Figures.zip!/Figure S3.pdf]

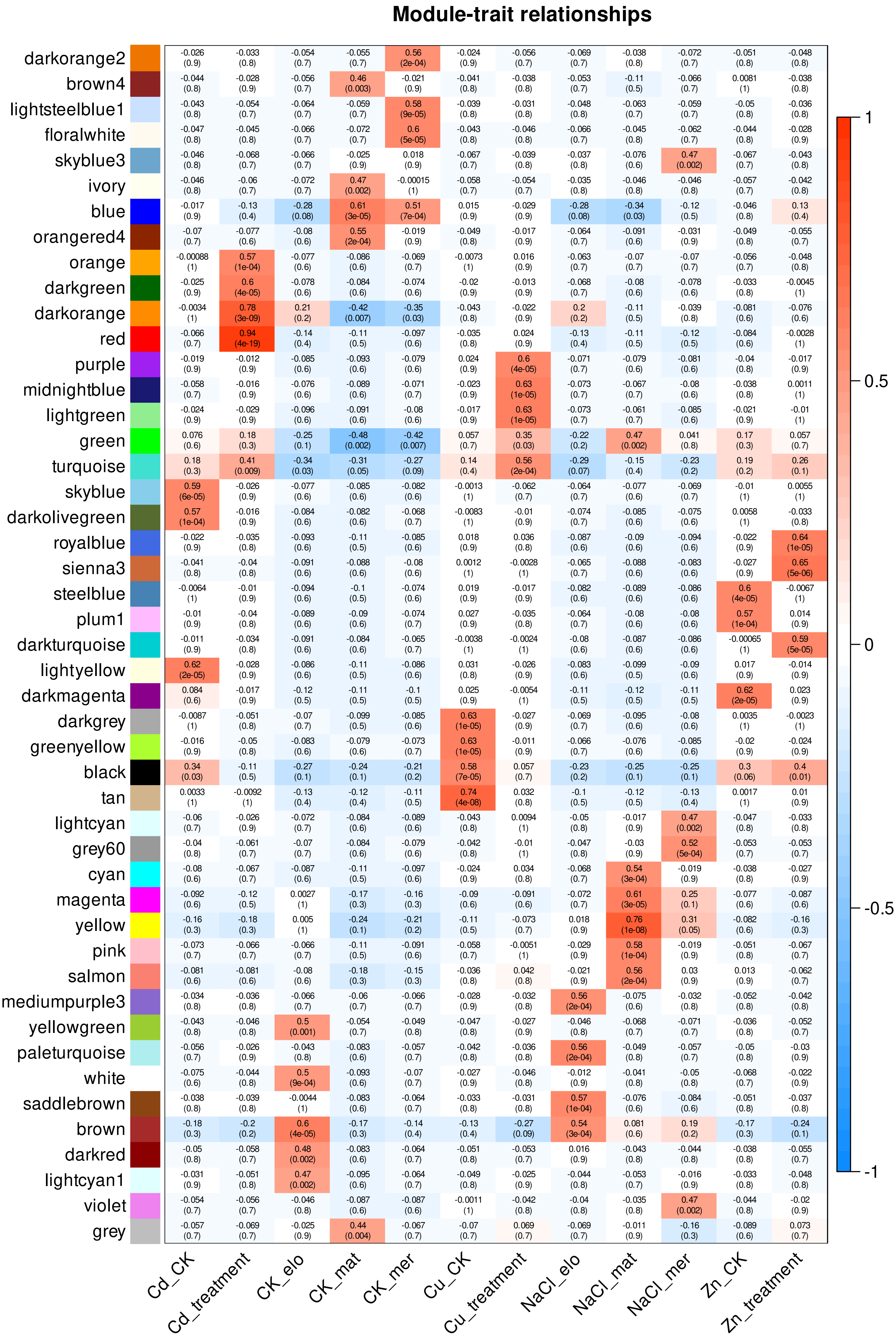
Module-trait relationships
darkorange2
brown4
lightsteelblue1
floralwhite
skyblue3
ivory
blue
orangered4
orange
darkgreen
darkorange
red
purple
midnightblue
lightgreen
green
turquoise
skyblue
darkolivegreen
royalblue
sienna3
steelblue
plum1
darkturquoise
lightyellow
darkmagenta
darkgrey
greenyellow
black
tan
lightcyan
grey60
cyan
magenta
yellow
pink
salmon
mediumpurple3
yellowgreen
paleturquoise
white
saddlebrown
brown
darkred
lightcyan1
violet
grey
Cd_CK
Cd_treatment
CK_elo
CK_mat
CK_mer
Cu_CK
Cu_treatment
NaCl_elo
NaCl_mat
NaCl_mer
Zn_CK
Zn_treatment
1
0.5
0
-0.5
-1
