# Supplementary material for: Genome-Wide Identification, Evolution and Expressional Analysis of OSCA Gene Family in Barley (Hordeum vulgare L.)
Source: Int J Mol Sci. 2022 Oct 27;23(21):13027. doi: 10.3390/ijms232113027 (PMC9653715; doi:10.3390/ijms232113027)
Supplement: Supplementary file 1 [file ijms-23-13027-s001.zip › Figures.zip!/Figure S4.pdf]

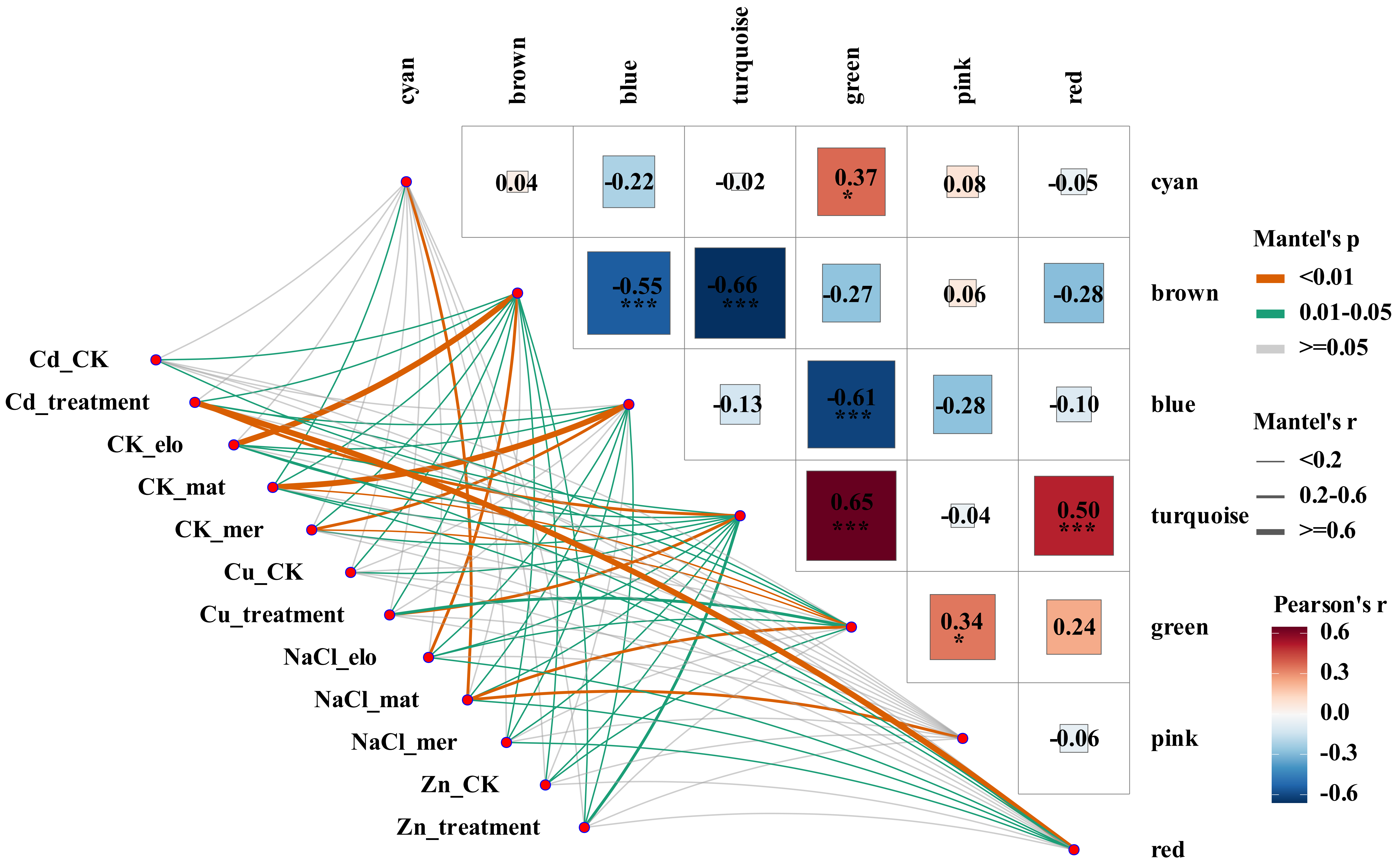

cyan
brown
blue
turquoise
green
pink
red
0.04
-0.22
-0.02
0.37 *
0.08
-0.05
-0.55 ***
-0.66 ***
-0.27
0.06
-0.28
-0.13
-0.61 ***
-0.28
-0.10
0.65 ***
-0.04
0.50 ***
0.34 *
0.24
-0.06
Cd_CK
Cd_treatment
CK_elo
CK_mat
CK_mer
Cu_CK
Cu_treatment
NaCl_elo
NaCl_mat
NaCl_mer
Zn_CK
Zn_treatment
Mantel's p
<0.01
0.01-0.05
>=0.05
Mantel's r
<0.2
0.2-0.6
>=0.6
Pearson's r
0.6
0.3
0.0
-0.3
-0.6
